# Comparison of Methodologies to Estimate Dietary Cadmium Intake in an Italian Population

**DOI:** 10.3390/ijerph17072264

**Published:** 2020-03-27

**Authors:** Tommaso Filippini, Kristen Upson, Giorgia Adani, Carlotta Malagoli, Claudia Baraldi, Bernhard Michalke, Marco Vinceti

**Affiliations:** 1 CREAGEN, Environmental, Genetic and Nutritional Epidemiology Research Center—Section of Public Health, Department of Biomedical, Metabolic and Neural Sciences, University of Modena and Reggio Emilia, 41125 Modena, Italy; tommaso.filippini@unimore.it (T.F.); giorgia.adani@unimore.it (G.A.); carlotta.malagoli@unimore.it (C.M.); 2 Department of Biomedical, Metabolic and Neural Sciences, University of Modena and Reggio Emilia, 41125 Modena, Italy; claudia.baraldi@unimore.it; 3 Department of Epidemiology and Biostatistics, College of Human Medicine, Michigan State University, East Lansing, MI 48824, USA; upsonkri@msu.edu; 4 Helmholtz Center Munich, German Research Center for Environmental Health GmbH, Research Unit Analytical BioGeoChemistry, 85764 Neuherberg, Germany; bernhard.michalke@helmholtz-muenchen.de; 5 Department of Public Health, Boston University, Boston, MA 02118, USA

**Keywords:** cadmium, food frequency questionnaire, biomarker, dietary intake, estimation

## Abstract

Cadmium is a metal that is toxic to humans, and the major source of cadmium exposure in the non-smoking general population is diet. To identify major food sources and lower exposure from diet, an accurate estimate of dietary cadmium intake is needed. Hence, the objectives of this study are to develop a method to assess dietary cadmium intake using a biomarker measurement and to improve the estimation of dietary cadmium intake when using a food frequency questionnaire (FFQ). In a random sample of an Italian population, we collected dietary habits by FFQ and measured cadmium in foods and beverages. These data were used to compute the estimated weekly dietary intake (WDI) of cadmium (µg) by kilogram (kg) of body weight (bw) (WDI_FFQ_). We also measured fasting serum cadmium levels by inductively-coupled plasma mass spectrometry. We used these data to develop a model for the estimation of the biomarker-derived dietary cadmium intake (WDI_bio_). In the 51 subjects recruited, the median level of serum cadmium was 0.041 µg/L (interquartile range (IQR): 0.030–0.054). The median WDI_FFQ_ and WDI_bio_ were 1.34 µg/kg bw/week (IQR: 0.86–1.70) and 0.72 µg/kg bw/week (IQR: 0.55–1.11), respectively. The correlation between the two estimates was low-to-moderate (r = 0.291). In exploratory analyses, the correlation was slightly higher in women and participants ages <50 years, and markedly higher in participants with body mass index <25 kg/m^2^ and smokers. Our approach allows for the dietary contribution to be isolated from the overall cadmium exposure measured with a biomarker; the estimated dietary cadmium intake was roughly similar to that estimated using the FFQ, especially in select subgroups. Future refinements to the biomarker-derived dietary cadmium intake approach should take into consideration additional sources of cadmium exposure, as well as factors affecting its absorption and metabolism.

## 1. Introduction

Cadmium is a toxic metal with both carcinogenic [1,2,3,4] and non-carcinogenic adverse effects [5,6,7] in humans. Smoking and pollution of ambient air from motorized traffic or industrial sources are major contributors to cadmium exposure [8,9]. In non-occupationally exposed non-smokers, the major source of cadmium is dietary intake [10]. Therefore, the characterization and reduction of cadmium exposure through diet in the general population are warranted from the public health perspective. The Joint FAO/WHO Expert Committee on Food Additives (JECFA) adopted the provisional tolerable monthly intake (PTMI) of 25 µg Cd/kg body weight/month [11], corresponding to a tolerable weekly intake (TWI) of approximately 6 µg Cd/kg body weight/week. The European Food Safety Authority established a lower tolerable weekly intake of 2.5 µg/kg body weight to ensure a high level of protection for all consumers, especially vulnerable subgroups [10]. Thus, an accurate estimate of dietary cadmium intake is needed to identify major food sources and to lower exposure from diet.

Dietary exposure to cadmium may be assessed through direct measurement of intake through foods or indirectly by biomonitoring [12]. However, these approaches have limitations. Dietary assessment of cadmium intake is considered prone to substantial measurement error of actual intake because of the large variation of cadmium content in food samples [13]. Biomonitoring of cadmium level is generally considered a valid method for assessment of exposure [14]. However, it captures exposure to cadmium from all sources, not just dietary; the specific contribution of dietary intake and inhalation to the overall exposure is not specified. For these reasons, it is not surprising that some studies have reported low correlations between biomarkers and dietary assessment of cadmium exposure [15,16]. Such a correlation has been particularly low among those with a substantial source of non-dietary exposure to cadmium—smokers.

The objectives of this study, carried out in an Italian population, are (1) to develop a method to assess dietary cadmium intake using a biomarker measurement, and (2) to improve the methods for the assessment of dietary cadmium intake by using a food frequency questionnaire. We also sought to provide a common estimate, the weekly dietary intake of cadmium by kilogram body weight, either biomarker-derived or based on the direct dietary assessment.

## 2. Methods

### 2.1. Study Population and Dietary Habits Assessment

The methods for the recruitment of the study population have already been reported in detail [17,18]. To summarize, we accessed the databases of the Modena Municipality General Registry Office. After approval from the local Ethics Committee, we recruited a random sample of individuals, ages 35–70 years, residing in Modena, a municipality of approximately 180,000 inhabitants located in the Emilia-Romagna region of Northern Italy. Specifically, we randomly selected a list of eligible individuals from each sex- and age-specific subgroup of the Modena adult population, using the “sample” routine of the Stata-11 statistical software (Stata Corp., College Station, TX, USA). We contacted 150 individuals by phone, asking for their participation in the study. Of those contacted, 34% (*n* = 51) agreed to participate. These participants were invited to attend a visit at a Modena Health Unit facility for the collection of a morning, fasting blood sample, and for the completion of two questionnaires. One questionnaire collected information on demographic and lifestyle characteristics. The other questionnaire was a food frequency questionnaire (FFQ) developed within the European Prospective Investigation into Cancer and Nutrition (EPIC) [19]. The EPIC–FFQ is a self-administered semi-quantitative FFQ; we used the version specifically developed and validated for the central–northern Italy population [20]. It was designed to estimate the frequency and amount of consumption of 188 food items over the previous year and used photos of serving sizes to ensure proper completion by participants. Data collected by the FFQ allowed for the assessment of nutrient and contaminant intakes.

### 2.2. Laboratory Analysis

We determined the cadmium content in foods characteristic of the usual diet of a northern Italian population and listed in the EPIC–FFQ questionnaire, as previously described. [17,21]. Briefly, we collected food samples from local stores and large retailers in Reggio Emilia and Modena provinces from October 2016 to February 2017. To prevent metal contamination from food containers, we used plastic tubes or jars and plastic cutlery during the collection and storage before analysis. We quantified cadmium in food using inductively coupled plasma-mass spectrometry with a limit of detection of 0.007 µg/kg [22].

We also quantified cadmium in biologic specimens from study participants. We collected fasting blood samples in a plastic tube, which were immediately centrifuged at 1000x *g* for 10 min and stored as serum aliquots of 1 mL at −15 °C until use. We measured serum cadmium levels using inductively coupled plasma–sector field–mass spectrometry (ICP–sf–MS) at the laboratory of the Helmholtz Center Munich German Research Center for Environmental Health (Research Unit Analytical BioGeoChemistry, Neuherberg, Germany) [8,23]. Specifically, we transferred 1 mL of frozen serum aliquot from each study participant on dry ice. We slowly thawed the samples in a refrigerator at 4 °C. We then vortexed and diluted samples 1:10 with Milli-Q water, which contained ^103^Rh as the internal standard. The final ^103^Rh concentration in the diluted serum samples was 1 µg/L. No HNO_3_ was added to serum samples as this would have precipitated proteins, resulting in the loss of analytes (Cd) and potentially clogging the ICP-nebulizer. We determined serum Cd levels using ICP–sf–MS (ELEMENT 2, Thermo Scientific, Bremen, Germany) for ^111^Cd determination in low-resolution mode. Sample introduction was carried out using an ESI-Fast-system (Elemental Scientific, Mainz, Germany) connected to a Micromist nebulizer with a cyclone spray chamber. The radio frequency (RF) power was set to 1200 W, the plasma gas was 15 L Ar/min, and the nebulizer gas was approximately 0.9 L Ar/min after daily optimization. The limit of quantification of the determination method was 18 ng/L related to native serum. Regular laboratory intercomparing studies (GEQUAS quality controls scheme and IRMM/EU certification campaign) as well as regular analysis of certificate material, namely ERM-BD-150 (skimmed milk powder from European Reference Materials-BD150), ERM-CD281 (rye grass from European Reference Materials-CD281), and Recipe RM ClinCal Plasma, were implemented for quality control [24,25,26]. The selection of CRMs was adopted according to sample matrix (e.g., plasma RM from Recipe Chemicals + Instruments GmbH, Munich, Germany) and to comparable (low) concentrations to samples (e.g., Cd in CRM CD281 or ERM-BD-150).

### 2.3. Data Analysis and Models for Dietary Cadmium Estimation

We developed two methodologies to estimate dietary cadmium intake. The first one was based on dietary assessment. We combined data on the cadmium content of foods and beverages with data on the dietary habits of the study population from the EPIC–FFQ, as previously reported [22]. Briefly, we multiplied element contents measured in food (µg/kg) with the intake of food and beverages (g/week), to estimate the daily dietary intake (DDI) of cadmium from the FFQ (1a). We then estimated the weekly dietary intake (WDI) by kilogram (kg) of body weight (bw), by multiplying the daily value per seven days, and dividing for the weight of participants (1b).

The equations for the estimation of dietary cadmium intake from FFQ were:

(1a) $${\mathrm{DDI}}_{\mathrm{FFQ}}\;\left(µg/\mathrm{day}\right)=\sum^{\;}\frac{\mathrm{Cd}\;\mathrm{food}\;\mathrm{content}\;\left(µg/\mathrm{kg}\right)\;\times \;\mathrm{food}\;\mathrm{intake}\;\left(g/\mathrm{day}\right)}{1000}$$

(1b) $${\mathrm{WDI}}_{\mathrm{FFQ}}\;\left(µg/\mathrm{week}\right){=\mathrm{DDI}}_{\mathrm{FFQ}}\;\left(µg/\mathrm{day}\right)\times \left(7\;\mathrm{days}/\mathrm{weight}\right)\;\;$$

The second methodology was based on biomarker levels, specifically the concentration of cadmium in serum. To estimate dietary cadmium from biomarker levels, we implemented the following steps:

1. We conducted linear regression, modeling serum levels of cadmium in relation to non-dietary determinants of cadmium concentration, namely, sex, age, and body mass index. From this regression model, we calculated the linear prediction of serum cadmium (sCd; Table S1).
2. We considered the ratio between total blood cadmium and circulated serum cadmium to be 10:1 (i.e., serum cadmium concentrations are considered to be 10% of whole-blood concentrations on average) [27].
3. We included both foods and beverages as dietary sources of cadmium intake. Based on previous studies [28,29,30], we set an average percentage of cadmium absorption of 5% in all participants after ingestion.
4. We adjusted serum cadmium concentrations to account for the contribution of smoking to cadmium exposure in participants with a heavy smoking habit and/or many years of smoking. First, we recoded smoking habits in the following manner. Former smokers were considered “non-smokers” if they quit more than 20 years before the date of sample collection. If they had quit in the past 20 years, former smokers were classified as “smokers” [31]. For this new category of “smokers”, we calculated the pack/year according to the NCI definition [32] and considered a pack to contain 20 cigarettes as generally sold in Italy [32]. In one case of cigarillos smoking, one cigarillo was considered equal to two cigarettes [33]. Second, we calculated the ratio (R) of pack-years of smoking to the total age of the participant. We estimated the adjusted serum cadmium concentration by multiplying the serum cadmium concentration by this ratio R and subtracting the product from the total serum cadmium levels.
5. Lastly, we used the following equations to derive the dietary cadmium intake from the predicted serum cadmium:

(2a) $${\mathrm{WDI}}_{\mathrm{bio}}=\left[\left(\mathrm{sCd}\;\times \;100/10\right)\;\times 100/5\right]\;\times \;\left(7\;\mathrm{days}/\mathrm{weight}\right)$$

(2b) $${\mathrm{WDI}}_{\mathrm{bio}}=\{[(\mathrm{sCd}\;-\;(\mathrm{sCd}\;\times \;R))\;\times 100/10]\;\times 100/5\}\;\times \;\left(7\;\mathrm{days}/\mathrm{weight}\right)$$

where WDI is the estimated weekly dietary intake for never smokers (2a) and for former and current smokers (2b); sCd is the predicted serum cadmium level based on the linear regression adjusted for age, sex and body mass index; R is the ratio of pack-years of smoking to the total age of the participants.

We performed the data analyses using Stata (version 15.2, Stata Corp., College Station, TX, USA). Data are generally presented using median and interquartile range (IQR). Pearson’s correlation (r) and its 95% confidence interval (CI) were estimated to assess the correlation between WDI_FFQ_ and WDI_bio_ in the overall population.

We conducted exploratory analyses in which we estimated the WDI_FFQ_, WDI_bio_, and their correlation in select subgroups of participants. We conducted these analyses to explore whether the estimation of dietary cadmium intake using dietary data and a biologic measurement were constant by sex, age (<50 and ≥50 years), body mass index (BMI) (<25 and ≥25 kg/m^2^), and smoking status. For these analyses, smoking status was considered in two ways. We analyzed smoking habits as collected using the questionnaire in three categories (never-, former-, and current-smokers). We repeated the analyses using two categories (non-smokers and smokers) considering the long half-life of cadmium in blood; former smokers were categorized as “non-smokers” if they had quit 20 years or more before the sample collection, and as “smokers” if less they had quit in the past 20 years.

## 3. Results

The characteristics of study participants are reported in Table 1. All the study participants (*n* = 51) were Caucasian with Italian citizenship. The age of the participants ranged between 35 and 71 years, with a median of 50 years (IQR: 44–62); 51% of participants were male. With regard to smoking habits, twenty-six individuals were never smokers. The sixteen former smokers and nine current smokers had similar median pack-years of smoking. Serum cadmium levels in the overall population were 0.041 (IQR: 0.030–0.054) µg/L, with slightly higher levels in participants younger than age 50 and those with a body mass index ≥25 kg/m^2^ (Table 1). Greater serum cadmium concentrations were also observed among current and former smokers compared to never smokers. The median WDI_FFQ_ and WDI_bio_ were 1.34 µg/kg bw/week (IQR: 0.86–1.70) and 0.76 µg/kg bw/week (IQR: 0.70–0.93), respectively (Table 2). A few participants (*n* = 3) had WDI_FFQ_ estimates that were greater than 2.5 µg/kg bw/week, the tolerable value established by the Food Safety Authority of the European Union [10]. The correlation between WDI_FFQ_ and WDI_bio_ was low-to-moderate (r = 0.291, 95% CI: 0.017 to 0.524) in the overall population. The WDI_FFQ_ estimate was higher than the WDI_bio_ estimate.

In the exploratory analyses, WDI_FFQ_ was higher in women (1.38 µg/kg bw/week, IQR: 0.86–1.91), participants ages 50 years and older (1.37 µg/kg bw/week, IQR: 0.87–1.69), those with BMI <25 (1.38 µg/kg bw/week, IQR: 0.86–1.99), and non-smokers (1.37 µg/kg bw/week, IQR: 0.86–1.74) (Table 2). With regard to WDI_bio_, it was similar between men and women and categories of BMI, and higher in participants ages <50 years (0.93 µg/kg bw/week, IQR: 77–1.07), and non-smokers (0.78 µg/kg bw/week, IQR: 73–1.32). A higher intake was observed for former smokers compared to never-smokers using WDI_FFQ_, but not WDI_bio_. The correlation between WDI_FFQ_ and WDI_bio_ was slightly higher in women (r = 0.352, 95% CI: −0.050 to 0.656) and participants <50 years (r = 0.370, 95% CI: −0.050 to 0.679), and markedly higher in participants with a BMI <25 (r = 0.555, 95% CI: 0.185 to 0.787). Correlations differed across smoking habit categories with higher correlation in smokers (r = 0.520, 95% CI: −0.014 to 0.824) and a lower correlation in non-smokers (r = 0.244, 95% CI: −0.087 to 0.625).

Sensitivity analyses using a cutpoint of 10 years since quitting smoking instead of 20 years for the categorization of non-smokers and smokers did not substantially change the results of WDI_FFQ_ and WDI_bio_; we observed similar values for non-smokers and smokers (Table S2). Using the 20-years categorization, we observed a WDI_FFQ_ of 1.37 µg/kg bw/week and a WDI_bio_ of 0.78 µg/kg bw/week. The corresponding estimates using the 10-years categorization were 1.17 and 0.68 µg/kg bw/week for WDI_FFQ_ and WDI_bio_, respectively. When we considered a lower absorption rate for cadmium (3%) in Equations (2a,b), estimates of dietary cadmium intake from the biomarker-derived estimate WDI_bio_ slightly increased. To reach the same level in serum of cadmium, a higher intake of cadmium is needed, thus the estimation of dietary intake from biomarker increases when the percentage of absorption decreases (Table S2).

## 4. Discussion

In this study, we sought to assess dietary cadmium exposure in an Italian population by comparing two methods to estimate it: one based on the modeling of serum cadmium levels and the other based on a validated food frequency questionnaire. Our results showed a generally low-to-moderate correlation between the two methodologies. In addition, we noted that the median WDI_FFQ_ was higher than the median WDI_bio_.

The low-to-moderate correlation between the two indicators may be due to several factors. First, we measured cadmium levels in serum instead of whole blood, using the information on the percent of cadmium bound to erythrocytes, which is generally 90% [27]. However, the binding of cadmium to metallothionein or to other plasma proteins may differ, and we did not account for these differences in our estimation of dietary cadmium intake using serum cadmium. Second, it is possible that a different rate of absorption of dietary cadmium may have affected the correlation between WDI_FFQ_ and WDI_bio_ [30]. However, the correlation between two WDIs was not altered in our sensitivity analysis using an alternative absorption rate. Third, we did not consider cadmium excretion in our estimation of WDI_bio._ Following dietary exposure, cadmium accumulates in the kidney and is slowly released into the urine. In subjects with kidney disease, excretion may be slower, thus increasing cadmium body burden [34]. In the present study, none of the study participants reported a history of major disease, including kidney disease, at the time of blood sampling. Therefore, the concern for kidney disease affecting the results is minimal. In addition, dietary exposure to cadmium does not appear high enough to increase the incidence of chronic kidney disease [35]. Fourth, other sources of cadmium may contribute to serum cadmium levels that were not accounted for in our estimation of the WDI_bio_ such as occupational exposure or inhalation of cadmium from indoor contaminants (such as passive smoking or cooking fumes), and outdoor air pollutants [8,36,37,38]. Fifth, we were not able to consider the status of other elements, for example, body iron stores, selenium, or other trace elements, which may interfere with cadmium absorption and metabolism. [30,39,40,41,42]. Lastly, the overestimation of the intake of foods that contribute to cadmium intake is possible. Foods generally considered healthy are more likely to be remembered, especially when people are confronted with dietary studies and nutritionist [43]. Since cereals and vegetables are the main source of cadmium in foods [22], it is possible that we overestimated the WDI_FFQ_.

In previous studies, the correlation between dietary exposure to cadmium based on dietary assessment and biomarker measurements have ranged from −0.10 to 0.61 [15,16,39,44,45,46,47,48,49,50]. Although two duplicate studies where urine samples were collected within 24 h of dietary assessment yielded a moderate correlation (0.40) [39,47], the correlation in other studies has been low [16,48,49]. This is likely due to the measurement of cadmium in urine characterizing long-term exposure (over several years or decades), whereas the dietary assessment captured recent dietary exposure. Our approach to disentangling the dietary contribution to cadmium exposure using biomarker measurements provides for a fairer comparison between biomarker measurement and dietary assessment.

Three prior studies to our knowledge have developed approaches to estimate cadmium intake from biomarkers. One study conducted in Japan, similar to our study, proposed a method using linear regression to estimate daily cadmium intake from biological sample levels. In that study, however, confounding by smoking seemed to be minimal to null, especially with regard to urinary cadmium levels [51]. When the linear regression models for the estimation of dietary cadmium from blood or urinary cadmium were applied to populations in East Asian countries, correlation coefficients above 0.7 were observed; the correlations were particularly high in female participants for whom the low smoking prevalence may have minimized confounding compared to males [52]. Another study with Swedish non-smoking women compared toxicokinetic models to estimate cadmium intake from urinary cadmium. The findings from that study suggest that, instead of an eight-compartment model based on many equations taking into account cadmium absorption, transport, and excretion [45], the use of a simpler one-compartment model based on kidney accumulation and excretion may adequately estimate cadmium intake [53]. Similar to the previous studies, we developed a method for the estimation of cadmium intake using linear regression and including non-dietary determinants (namely age, sex, and body mass index) in the model. We also attempted to account for smoking habits by developing different equations for smokers and non-smokers.

A strength of our study is the estimation of WDI_FFQ_ using a validated food frequency questionnaire (FFQ) version that was developed and validated for the central–northern Italy population [20]. This was complemented by our measurement of cadmium in foods commonly consumed by the study population [22]. Our study additionally benefitted from the recruitment of a representative sample of residents from the Modena municipality [8,54]. The characteristics of our study population are similar to those of the overall Italian and regional population [55,56], strengthening the external validity of our results. Despite these strengths, this study was limited by the small sample size and limited age range (35–70 years). This resulted in less precise estimates, and, therefore, replication of this study in a larger study population is warranted.

Contamination of food and beverages from toxic metals like cadmium represents an important public health issue, especially in heavily contaminated areas [12]. Although staple foods generally contribute to only 40–60% of total dietary cadmium intake, dietary cadmium exposure can occur from the intake of food with substantial cadmium content, such as seafood, mushrooms, offal, and chocolate [4,22,57,58,59,60,61]. Avoidance or limitation of consumption of these foods, especially in vulnerable populations like children, pregnant women, or elderly people, is highly advisable [12,62]. Therefore, an adequate assessment of their dietary intake is of utmost importance, as well as the ability to compare actual exposure with the safety values set by international agencies [10,60]. It should be noted that in our study, some participants had WDI_FFQ_ estimates that exceeded the tolerable value of 2.5 µg/kg bw/week established by the Food Safety Authority of the European Union [10].

## 5. Conclusions

In this study, we present an approach to isolate the dietary contribution to cadmium exposure from a biomarker measurement, accounting for smoking habits. The biomarker-based approach yielded roughly similar estimates of the weekly dietary intake of cadmium to that derived from dietary assessment by an FFQ. Despite the study limitations, our methods could be useful in a scenario in which the estimation of dietary cadmium intake is sought for risk assessment, but only a biomarker of cadmium is available. Future refinements to this modeling should take into consideration additional sources of cadmium exposure, as well as factors affecting its absorption and metabolism.

## Figures and Tables

**Table 1:** Characteristics of study population and cadmium serum median (interquartile range—IQR) levels (µg/L).

Characteristics	*n*	(%)	Median	IQR
*All subjects*	51	(100)	0.041	0.030–0.054
*Sex*				
Men	26	(51)	0.040	0.032–0.055
Women	25	(49)	0.041	0.030–0.054
*Age*				
<50 years	23	(45)	0.043	0.036–0.059
≥50 years	28	(55)	0.036	0.028–0.047
*Education*				
Primary school	3	(6)	0.027	0.023–0.105
Middle school	10	(20)	0.040	0.030–0.048
High school	23	(45)	0.039	0.029–0.049
College or more	15	(29)	0.045	0.035–0.059
*Occupation*				
Industrial sector ^a^	6	(12)	0.044	0.037–0.059
Services ^b^	27	(53)	0.043	0.031–0.054
Retired	11	(21)	0.037	0.020–0.058
Not employed	7	(14)	0.036	0.030–0.048
Body mass index	25.2	(23.0–28.4)		
<25 kg/m^2^	23	(45)	0.038	0.029–0.048
≥25 kg/m^2^	28	(55)	0.043	0.031–0.061
*Smoking habits as reported*				
Never smokers	26	(51)	0.036	0.029–0.043
Former smokers	16	(31)	0.045	0.032–0.053
Current smokers	9	(18)	0.055	0.042–0.059
Pack/year ^c^	14.2	(6.8–19.0)		
Former smokers ^c^	15.0	(5.5–19.5)		
Current smokers ^c^	13.5	(7.2–14.3)		
*Smoking habits recoded* ^d^				
Non-Smokers	37	(73)	0.037	0.030–0.046
Smokers	14	(27)	0.051	0.041–0.059

^a^ Industrial sector includes engineering workers and chemical workers; ^b^ Health, education, and business; ^c^ Median (IQR); ^d^ Smoking habits recoded with former smokers categorized as “non-smokers” if they quit 20 years or more before sample collection, and as “smokers” if they quit less than 20 years before sample collection.

**Table 2:** Estimated weekly dietary intake (median (50th) and interquartile range (IQR) reported in µg per kilograms of body weight per week) using food frequency questionnaire (WDI_FFQ_) and serum cadmium biomarker levels (WDI_bio_) in select subgroups, and Pearson’s correlation (r) with 95% confidence interval (CI) between WDI_FFQ_ and WDI_bio_.

Subgroups		WDI_FFQ_			WDI_bio_		Correlation WDI_FFQ_–WDI_bio_	
	50th	(IQR)	Range	50th	(IQR)	Range	r	(95% CI)
*All subjects*	1.34	0.86–1.70	0.26–3.18	0.76	0.70–0.93	0.33–1.32	0.291	(0.017, 0.524)
*Sex*								
Men	1.30	(0.87–1.63)	0.26–3.18	0.76	(0.70–0.93)	0.33–1.08	0.214	(−0.189, 0.556)
Women	1.38	(0.86–1.91)	0.30–3.07	0.77	(0.70–0.93)	0.33–1.32	0.352	(−0.050, 0.656)
*Age*								
<50 years	1.29	(0.80–2.15)	0.26–3.07	0.93	(0.77–1.07)	0.41–1.32	0.370	(−0.050, 0.679)
≥50 years	1.37	(0.87–1.69)	0.30–3.18	0.73	(0.69–0.77)	0.33–0.93	0.233	(−0.154, 0.557)
*Body mass index*								
<25 kg/m^2^	1.38	(0.86–1.99)	0.59–3.07	0.78	(0.70–0.93)	0.33–1.23	0.555	(0.185, 0.787)
≥25 kg/m^2^	1.21	(0.84–1.62)	0.26–3.18	0.75	(0.71–0.92)	0.41–1.32	0.112	(−0.273, 0.465)
*Smoking habits*								
Never-smokers	1.27	(0.73–1.60)	0.26–3.07	0.86	(0.75–0.99)	0.62–1.32	0.371	(−0.020, 0.663)
Former-smokers	1.66	(1.24–1.95)	0.62–3.18	0.75	(0.71–0.80)	0.33–0.93	0.416	(−0.101, 0.756)
Current-smokers	1.17	(1.03–1.36)	0.72–1.55	0.68	(0.59–0.74)	0.33–0.96	0.406	(−0.354, 0.843)
*Smoking habits recoded* ^a^								
Non-smokers	1.37	(0.86–1.74)	0.26–3.18	0.78	(0.73–1.32)	0.33–1.32	0.244	(−0.087, 0.625)
Smokers	1.24	(1.03–1.55)	0.62–2.32	0.68	(0.59–0.74)	0.33–0.96	0.520	(−0.014, 0.824)

^a^ Smoking habits recoded with former smokers categorized as “non-smokers” if they quit 20 years or more before sample collection, and as “smokers” if they quit less than 20 years before sample collection.

## Supplementary Materials

The following are available online at https://www.mdpi.com/1660-4601/17/7/2264/s1, Table S1: Linear regression coefficients (Beta) and 95% confidence intervals (CI) between serum cadmium and relevant confounders for adjustment levels, Table S2: Estimated weekly dietary intake (median (50th) and interquartile range (IQR) reported in µg per kilograms of body weight per week) using food frequency questionnaire (WDI_FFQ_) and using serum cadmium biomarker levels (WDI_bio_) in total population and in select subgroups considering a value of 3% for cadmium absorption in Equations (2a,b).
