# Supplementary material for: Comparison of Methodologies to Estimate Dietary Cadmium Intake in an Italian Population
Source: Int J Environ Res Public Health. 2020 Mar 27;17(7):2264. doi: 10.3390/ijerph17072264 (PMC7177715; doi:10.3390/ijerph17072264)
Supplement: Supplementary file 1 [file ijerph-17-02264-s001.pdf]

Table S1. Linear regression coefficients (Beta) and 95% confidence intervals (CI) between serum cadmium and relevant confounders for adjustment levels.

	Beta Coefficients	(95% CI)
age	−0.57	(−1.20 to 0.06)
sex	−7.71	(−20.73 to 5.30)
body mass index	1.03	(−0.36 to 2.43)

Table S2. Estimated weekly dietary intake (median (50th) and interquartile range (IQR) reported in μg per kilograms of body weight per week) using food frequency questionnaire (WDI_{FFQ}) and using serum cadmium biomarker levels (WDI_{bio}) in total population and in selected subgroups considering a value of 3% for cadmium absorption in equations (2a) and (2b).

Subgroups	WDI_{FFQ}			WDI_{bio}		
	50th	(IQR)	Range	50th	(IQR)	Range
All subjects	1.34	(0.86–1.70)	0.26–3.18	0.84	(0.73–1.08)	0.55–1.60
Sex						
Men	1.30	(0.87–1.63)	0.26–3.18	0.83	(0.75–0.98)	0.55–1.60
Women	1.38	(0.86–1.91)	0.30–3.07	0.84	(0.72–1.23)	0.55–1.48
Age						
<50 years	1.29	(0.80–2.15)	0.26–3.07	0.99	(0.92–1.23)	0.68–1.60
≥50 years	1.37	(0.87–1.69)	0.30–3.18	0.75	(0.70–0.81)	0.55–1.48
BMI [a]						
<25	1.38	(0.86–1.99)	0.59–3.07	0.93	(0.77–1.08)	0.55–1.48
≥25	1.21	(0.84–1.62)	0.26–3.18	0.77	(0.73–1.05)	0.59–1.60
Smoking habits						
Never-smokers	1.27	(0.73–1.60)	0.26–3.07	0.86	(0.75–0.99)	0.62–1.32
Former-smokers	1.66	(1.24–1.95)	0.62–3.18	0.76	(0.73–0.89)	0.55–1.48
Current-smokers	1.17	(1.03–1.36)	0.72–1.55	1.14	(0.98–1.23)	0.55–1.60
Smoking habits (recoded)						
Non-smokers	1.37	(0.86–1.74)	0.26–3.18	0.81	(0.73–0.96)	0.55–1.48
Smokers	1.24	(1.03–1.55)	0.62–2.32	1.14	(0.98–1.23)	0.55–1.60
